# Dysentery in California

**Published:** 1852-06

**Authors:** 


The climate of California, that *hotbed of Dysentrry and Diarrhœa*, is eminently calculated for the production of this disease. While in that country, I not unfrequently saw the thermometer stand, during a part of the day, as high as 109° Fahr.; and on one occasion, as high as 113° 58, and then, before the following morning, drop down below 60°. Hence, I must regard the prevalence of the disease there, with the unacclimated, as the relation of cause and effect. In fact, so common was diarrhea and dysentery in that country, that a natural stool was rarely seen. With these facts presenting themselves, and in the absence of a better cause, we must regard the cool and variable autumn, succeeding a hot, dry summer, as the chief cause of the epidemic.—*N. O. Med. Jour.*

